# A Rare Case of Methamphetamine-Induced Diffuse Gastrointestinal Ischemia

**DOI:** 10.7759/cureus.76857

**Published:** 2025-01-03

**Authors:** Nicole Johnsen, Andrew Chang, Kelley Chuang, Satya Patel, Simon Wu

**Affiliations:** 1 Internal Medicine, David Geffen School of Medicine at the University of California, Los Angeles, Los Angeles, USA; 2 Internal Medicine, Veterans Affairs Greater Los Angeles Healthcare System, Los Angeles, USA

**Keywords:** clinical methamphetamine effects, drug-induced ischemia, extensive gi ulceration, gastrointestinal ischemia, methamphetamine, rare cause of acute abdominal pain, substance use

## Abstract

Methamphetamine is a widely used substance known for cardiovascular and neurological complications; however, its gastrointestinal effects remain poorly understood. While rare, methamphetamine-induced gastrointestinal ischemia has high morbidity and mortality rates, with limited case reports in the literature. We present a case of a 48-year-old man with a history of gastroesophageal reflux disease, alcohol use disorder in remission, and previously documented methamphetamine use who presented with two weeks of episodic abdominal pain, nausea, and hematemesis. Significant laboratory and imaging findings included acute anemia, urine toxicology confirming the presence of amphetamines, and computed tomography imaging showing wall thickening in the distal esophagus and stomach. On endoscopy, he was found to have diffuse ulcerations in the distal esophagus and post-pyloric region with pathology indicative of methamphetamine-induced gastrointestinal ischemia. This case highlights the importance of considering amphetamine-induced gastrointestinal ischemia in patients with stimulant use disorders who present with acute abdominal symptoms. Early recognition of this etiology can guide targeted counseling and management to improve outcomes.

## Introduction

Methamphetamine, a potent central nervous system stimulant, is known for its severe effects on various organ systems. While its cardiovascular and cerebrovascular complications, including hypertension, myocardial infarction, and stroke, are well-established [1], its impact on the gastrointestinal (GI) tract remains poorly understood. Although rare, methamphetamine use has been associated with widespread GI ischemia, a potentially life-threatening condition that demands prompt recognition and management to prevent severe complications.

Unlike conventional GI ischemia caused by embolism, thrombosis, or vasoconstriction [2], methamphetamine-induced ischemia is particularly concerning due to its diffuse and multifactorial ischemic effects. Methamphetamine induces extensive GI ischemia through mechanisms such as potent splanchnic vasoconstriction, necrotizing vasculitis, cardiovascular shock, and sympathomimetic vasospasm [3]. The precise contributions of these mechanisms to bowel ischemia remain unclear; however, the potential for severe complications, including bowel necrosis, perforation, and sepsis, is significant, leading to high morbidity and mortality rates [4,5].

Despite the severity of this condition, there are few documented cases in the literature [3-8], highlighting the need for healthcare providers to have an increased index of suspicion for this illness. This report contributes to the limited body of knowledge on this rare condition by presenting a case of diffuse GI ischemia secondary to methamphetamine use.

## Case presentation

We report a rare case of diffuse GI ischemia secondary to methamphetamine use in a 48-year-old Caucasian male who presented to the emergency department with a two-week history of episodic postprandial abdominal pain. The patient experienced acute worsening of his abdominal pain following a large meal, accompanied by nausea and hematemesis. He also reported an unintentional weight loss of 20 pounds over the past year. His medical history was notable for gastroesophageal reflux disease, alcohol use disorder in remission, and previously documented inhaled and intranasal methamphetamine use. Importantly, he had no known history of prior vascular events or vasculopathies.

As shown in Table 1, laboratory studies revealed decreasing hemoglobin over the course of hospitalization, raising concern for acute blood loss from the reported hematemesis. Although the patient stated he had not intentionally used amphetamine recently, urine toxicology and confirmatory testing were positive for amphetamines on admission.

**Table 1:** Notable laboratory findings. Notable laboratory findings during hospital days one to three. An acute hemoglobin drop of approximately 2 g/dL was observed between days one and three. On day one, the urine drug screen was positive for amphetamines, with confirmatory testing revealing elevated levels of methamphetamine and amphetamine. MDEA: methylenedioxyethylamphetamine; MDMA: methylenedioxymethamphetamine; MDA: methylenedioxyamphetamine.

Parameter	Value (day 1)	Value (day 2)	Value (day 3)	Normal value (reference range)
Hemoglobin (g/dL)	15.5	14.3	13.4	13.5-17.5
Qualitative urine toxicology screen (ng/mL)	Amphetamines: Positive	-	-	<500
	Benzodiazepines: Negative			<300
	Buprenorphine: Negative			<10
	Cannabinoids: Negative			<50
	Cocaine: Negative			<150
	Fentanyl: Negative			<1
	Methadone: Negative			<300
	Opiates: Negative			<100,000
	Ethanol: Negative			<5,000
Quantitative urine toxicology reflex panel for amphetamines (ng/mL)	Methamphetamine: Positive (988)	-	-	<500
	Amphetamine: Positive (776)			<500
	MDEA: Negative			<500
	MDMA: Negative			<500
	MDA: Negative			<500
	Phentermine: Negative			<500

**Figure 1:**
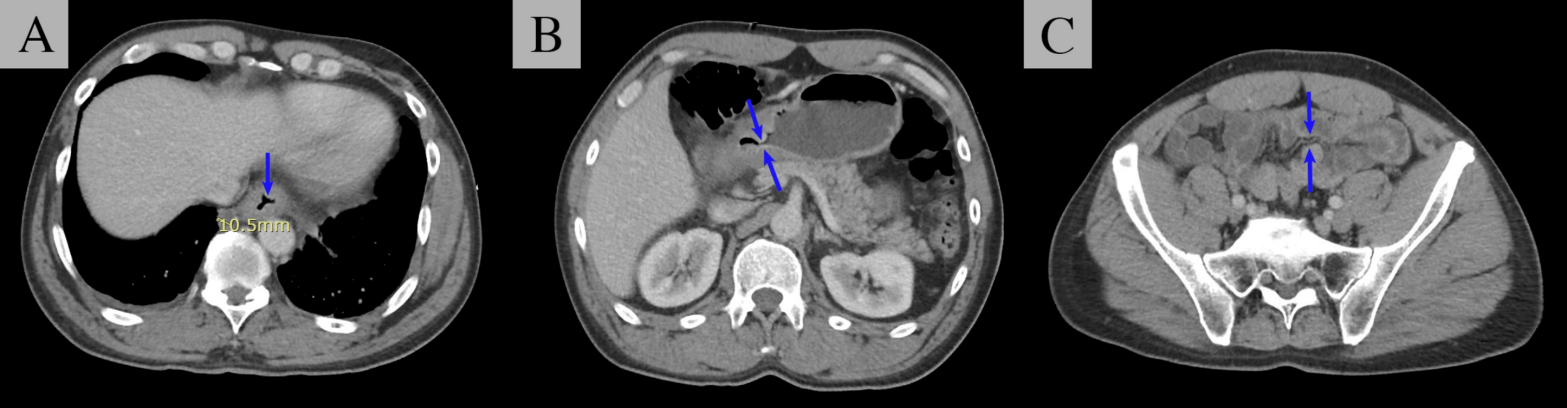
Contrast-enhanced CT of the abdomen and pelvis. CT imaging revealed nonspecific thickening of the distal esophageal (A), irregular thickening of the distal stomach (B), and a focal area of increased soft tissue density with luminal narrowing in the mid-transverse colon (C). All abnormal findings are indicated by blue arrows.

A contrast-enhanced CT of the abdomen and pelvis revealed wall thickening in the distal esophagus (Figure 1A) and stomach (Figure 1B), along with luminal narrowing of the mid-transverse colon (Figure 1C). Due to the concerning finding of colonic luminal narrowing and the patient’s history of weight loss, the patient was admitted for expedited evaluation of potential colonic malignancy.

**Figure 2:**
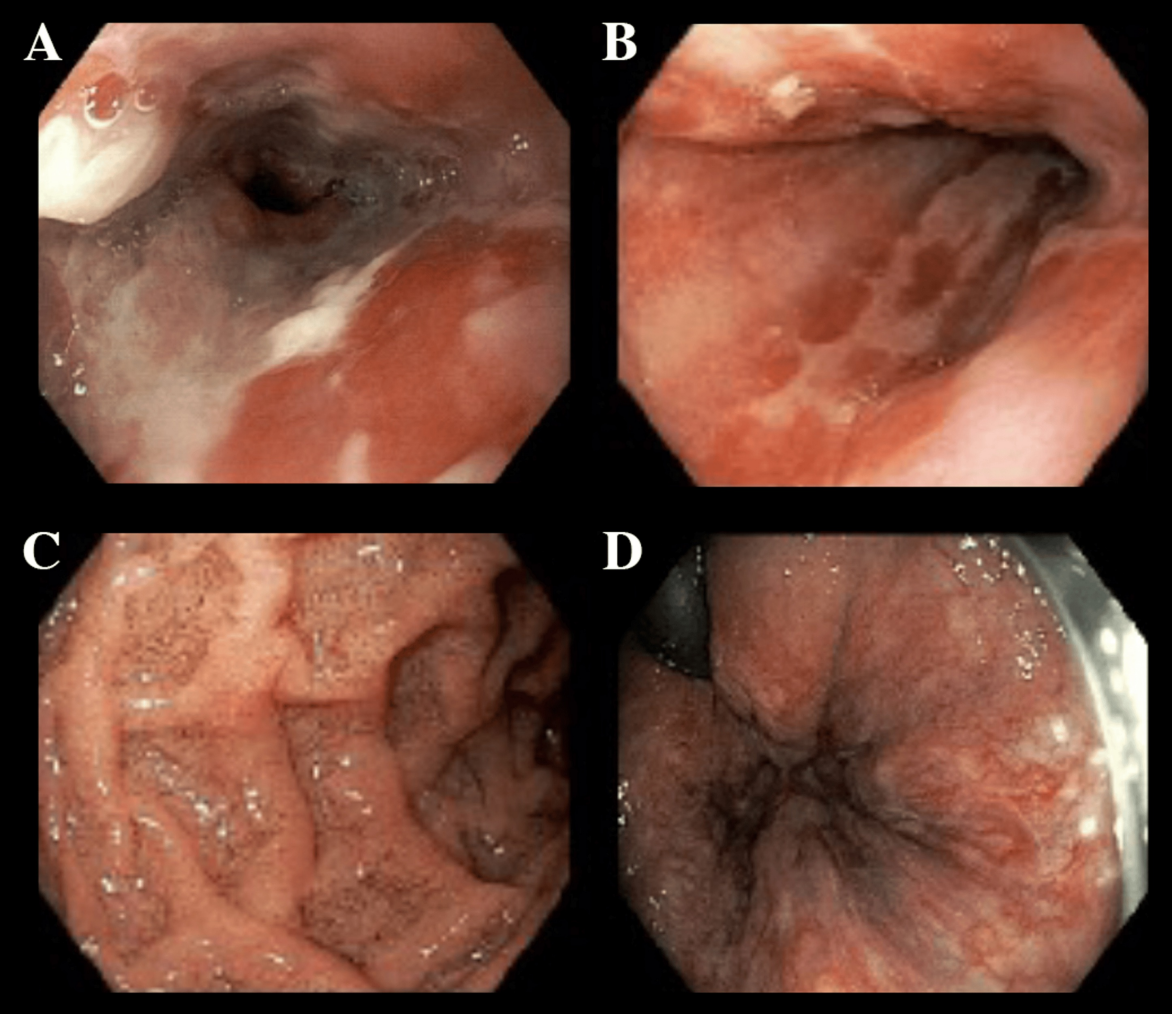
Endoscopic visualization of the upper and lower gastrointestinal tract. Upper endoscopy identified ulcerations in the distal esophagus (A) and similar erosive changes in the post-pyloric region of the stomach (B), accompanied by diffusely pale gastric mucosa (C). Lower endoscopy revealed widespread pallor of the colonic mucosa, most prominent at the splenic flexure (D). Collectively, these findings are consistent with diffuse ischemic changes in the gastrointestinal tract.

Subsequent endoscopic investigations, including esophagogastroduodenoscopy (EGD) and colonoscopy, revealed diffuse ulcerations in the distal esophagus (Figure 2A) and post-pyloric regions (Figure 2B). Additionally, they showed diffusely pale mucosa in the stomach (Figure 2C) and colon, with the most pronounced changes observed at the splenic flexure (Figure 2D).

Importantly, no masses or strictures were identified on colonoscopy, decreasing immediate concern for neoplasm. Histopathological examination from biopsies taken during the EGD revealed chronic inflammation, intestinal metaplasia, and active inflammation in the distal esophagus, stomach, and post-pyloric regions, without evidence of dysplasia or malignancy. Immunohistochemical staining for *Helicobacter pylori *was positive at the gastroesophageal junction but negative in the antral ulcer (Table 2). No biopsies were obtained from the colon.

**Table 2:** Helicobacter pylori status of endoscopic biopsy specimens. *Helicobacter pylori *status was determined via histological examination of hematoxylin and eosin-stained tissue from endoscopic biopsies. Tissue from the gastroesophageal junction tested positive for* H. pylori*, while tissue from the antral ulcer tested negative.

Biopsy location	*H. pylori* result
Gastroesophageal junction	Positive
Antral ulcer	Negative

The patient’s clinical presentation, history of methamphetamine use, absence of prior vasculopathy, and the diffuse nature of his ischemic changes on endoscopy strongly implicated methamphetamine-induced GI ischemia as the primary etiology. The patient was counseled to avoid stimulant use, prescribed quadruple therapy for *H. pylori*, and scheduled for a follow-up EGD in two months to reassess mucosal healing and confirm response to treatment. Unfortunately, this patient was lost to follow-up, and there has been no repeat EGD performed.

## Discussion

This case highlights the diagnostic challenges and clinical severity of methamphetamine-induced gastrointestinal ischemia, a rare yet potentially life-threatening condition [4,5]. The clinical presentation is often nonspecific, with patients commonly exhibiting abdominal pain, nausea, vomiting, and weight loss, symptoms that can easily be misattributed to a variety of other gastrointestinal conditions [3]. This clinical overlap often leads to significant diagnostic delays. Without prompt intervention, methamphetamine-induced gastrointestinal ischemia can progress to more severe manifestations, including bowel necrosis and ulceration, which may present as hematemesis or hematochezia [4]. These complications can ultimately advance to bowel perforation and sepsis, which are associated with high morbidity and mortality [4,5]. In addition, chronic methamphetamine use can disrupt intestinal motility, increasing the risk of conditions such as pseudo-obstruction or ileus, which can closely mimic the presentation of bowel obstruction, further complicating the diagnosis [9].

The presence of hematemesis suggested an upper gastrointestinal bleed, common causes of which include peptic ulcer disease, Mallory-Weiss tears, and gastritis [10]. The colicky abdominal pain raised concerns for embolic mesenteric ischemia, bowel obstruction, or biliary colic, all of which can present with similar clinical manifestations [2,11,12]. Additionally, the significant weight loss prompted suspicion of gastrointestinal malignancy, including gastric or colorectal cancer, and raised the possibility of chronic inflammatory conditions like Crohn’s disease [13,14]. The nonspecific nature of these symptoms posed a considerable diagnostic challenge, prompting a comprehensive workup. Initial CT imaging revealed thickening of the esophageal, gastric, and colonic walls, which raised further concerns for malignancy and resulted in the patient’s admission for an expedited diagnostic workup.

The diagnosis of methamphetamine-induced gastrointestinal ischemia is established through a combination of clinical history, imaging studies, and endoscopic evaluation. In patients presenting with nonspecific gastrointestinal symptoms, a thorough social history and chart review are essential to assess for any documented substance use [3,6]. Imaging studies, such as abdominal CT, should be employed to evaluate for malignancy, bowel wall thickening, and obstruction, all of which may mimic or contribute to gastrointestinal symptoms [3,5,7]. CT angiography may also be considered to assess for vascular etiologies [3]. Given the high mortality rate of methamphetamine-induced gastrointestinal ischemia and the need for prompt intervention, it must always be included in the differential diagnosis for patients with nonspecific gastrointestinal symptoms and a history of chronic methamphetamine use. Definitive diagnosis is confirmed through upper and lower endoscopy, which typically reveals diffuse mucosal ischemia, edema, and, in more advanced cases, ulceration, hemorrhage, or bowel necrosis [4,7].

In this case, endoscopy revealed diffuse gastrointestinal ischemia and ulceration without evidence of masses or strictures, effectively ruling out gastrointestinal malignancy and suggesting a vascular etiology. Although *H. pylori* infection could account for some localized inflammation and ulceration, the extensive ischemic changes more strongly indicated a vascular cause affecting multiple territories, prompting the clinical team to consider drug-induced ischemia. A thorough chart review ultimately uncovered the patient’s history of inhaled and intranasal methamphetamine use, which confirmed the diagnosis. Early identification of methamphetamine as the likely cause allowed for timely intervention and counseling on stimulant cessation, reducing the risk of further complications.

If no complications are present, management is generally conservative, focusing on fluid resuscitation, electrolyte balance, and counseling on substance cessation to prevent recurrence and further complications [7,8], as it was in our case. In more severe cases, surgical intervention may be necessary, particularly for bowel necrosis or perforation, which often requires resection of the affected segment [3,6]. In cases of bowel perforation with peritoneal contamination, IV antibiotics are critical to prevent severe infection and sepsis [5]. Post-surgery, patients must be closely monitored for signs of infection and the resolution of bowel function [3,5,6]. Additionally, repeat endoscopy is typically recommended to assess for ischemia resolution [7].

Overall, this case report adds to the existing literature by offering valuable insights into the diagnostic challenges, clinical presentation, and diagnosis of this rare condition [3-8]. It highlights the critical need for clinicians to maintain a high index of suspicion for methamphetamine-induced gastrointestinal ischemia in patients with nonspecific gastrointestinal symptoms and a history of chronic methamphetamine use. Finally, it emphasizes the importance of early recognition and intervention to prevent potential complications.

## Conclusions

In summary, healthcare providers must maintain a heightened level of suspicion for methamphetamine-induced gastrointestinal ischemia in patients with nonspecific gastrointestinal symptoms and a known history of chronic methamphetamine use. This diagnosis becomes particularly important when a patient in this population presents with colicky abdominal pain and hematemesis, especially after ruling out more common gastrointestinal conditions such as peptic ulcer disease or malignancy. A comprehensive social history, including both intentional and unintentional stimulant use, plays a critical role in guiding accurate diagnosis and distinguishing this condition from other potential causes. Additionally, diffuse ischemic changes identified during upper and lower endoscopies should heighten clinical suspicion for this rare diagnosis. Early identification and intervention are essential to prevent complications such as bowel necrosis, perforation, and sepsis. Upon diagnosis, it is crucial to counsel the patient on substance use cessation and schedule a repeat endoscopy to monitor the improvement of mucosal ischemia. By including methamphetamine-induced ischemia in the differential diagnosis, clinicians can ensure timely diagnosis and treatment, ultimately reducing morbidity and mortality associated with this condition.
